# Mechanisms regulating expression of the HPV 31 L1 and L2 capsid proteins and pseudovirion entry

**DOI:** 10.1186/1743-422X-4-19

**Published:** 2007-02-26

**Authors:** Patrick L Hindmarsh, Laimonis A Laimins

**Affiliations:** 1 Department of Microbiology – Immunology, Feinberg School of Medicine, Northwestern University, Chicago, Illinois, 60611, USA; 2 Louisiana State University Health Sciences Center, Department of Microbiology, Immunology and Parasitology 1901 Perdido St. New, Orleans, Louisiana 70112, USA

## Abstract

Human papillomaviruses (HPV) infect stratified epithelia and restrict expression of late capsid genes to highly differentiated cells. In order to begin to understand the processes regulating HPV 31 infection we examined the synthesis of the HPV 31 capsid proteins, L1 and L2, using heterologous expression systems. Similar to studies in HPV 16, expression of wild type HPV 31 L1 and L2 from heterologous promoters resulted in very low levels of synthesis. In contrast, modification of the codons in the capsid genes to ones more commonly used in cellular genes resulted in high-level synthesis. Through the use of chimeric proteins that fused fragments of wild type L1 to Green Fluorescent Protein (GFP) coding sequences, a short region was identified that was sufficient to inhibit high level synthesis and similar elements were detected in L2. One element was localized to the 3' end of the L1 gene while a series of elements were localized at the 3' end of the L2 coding sequences. These observations are most consistent with negative RNA regulatory elements controlling the levels of L1 and L2 synthesis that are distinct from those identified in HPV 16. Expression vectors for the codon modified HPV 31 capsid proteins were then transfected together with GFP reporter plasmids to generate HPV 31 pseudoviruses. Infection of cells with HPV 31 pseudoviruses in the presence of the inhibitors, chlorpromazine, nystatin or methyl-beta-cyclodextrin, demonstrated that HPV 31, like HPV 16, enters human and monkey cells through a clathrin-mediated pathway rather than through caveolae as previously reported. This suggests that high-risk HPV types may enter cells through common mechanisms.

## Background

Papillomavirus are non-enveloped, small double-stranded DNA viruses that target epithelial tissue for infection [1]. More than one hundred different types of human papillomaviruses (HPV) have been identified [2] and approximately one third of these types infect the anogenital epithelium. Infections by genital papillomaviruses are one of the most common sexually transmitted viral infections. The viruses that infect the genital tract are further classified as either high risk or low risk viruses. Low risk viruses induce benign warts that rarely progress to malignancy. In contrast, infections by high-risk HPV types can lead to the development of cervical cancer which is one of the most common cancers worldwide [1,3,4].

Infection by HPV's is believed to occur following micro-traumas of the epithelia that expose the basal cells to entry [1,3]. Following entry into cells, the viral genomes are established as nuclear plasmids. Infected cells in the stratum basale maintain viral genomes at approximately 50 copies and transcription is limited to the early genes [4]. Expression of early transcripts is directed primarily from a promoter upstream of E6 and the early gene products regulate viral gene expression, viral genome replication and cellular transformation.

The productive phase of the papillomavirus life cycle is keyed to the differentiation program of the infected keratinocytes [4]. As cells in the basal layer divide, one of the daughter cells migrates towards the upper epithelial layers. Normal uninfected epithelial cells exit the cell cycle as they leave the basal layer and begin a program of terminal differentiation. In contrast, HPV infected cells remain active in the cell cycle through the action of HPV early genes E6 and E7. A subset of infected suprabasal cells re-enter S-phase and amplify viral genome copy numbers to thousands of copies per cell. Concomitant with genome amplification is activation of the late promoter which directs expression of the E1^E4, E5 late proteins as well as the capsid proteins L1 and L2. Expression of capsid proteins is restricted to differentiated epithelial cells and is regulated by both transcriptional and post-transcriptional mechanisms.

HPV virions have been produced in the laboratory through the use of organotypic raft cultures [5]. These cultures allow for the growth of cells at the air-liquid interface, resulting in differentiation of the keratinocytes. When epithelial cells that maintain HPV episomes are grown in rafts, virus production occurs in the upper strata [5-7]. The titers of virus produced from these cultures are low and this has limited the use of these reagents for investigating the papillomavirus infectious life cycle [8]. Alternative methods to study HPV infection include the use of viral-like particles (VLPs) as well as pseudoviruses which encapsidate reporter genes inside VLPs [9-11].

The mechanisms that regulate HPV entry into cells are beginning to be elucidated. Initial binding of HPVs is thought to occur on many cell types through an association with heparin sulfate and this is likely followed by engagement of additional receptor proteins on the cell surface [12-14]. Many DNA viruses enter cells through one of two dynamin-dependent pathways involving use of caveolae or clathrin coated pits [15,16]. Caveolae are invaginations of the plasma membrane that are associated with lipid rafts and are stabilized by the presence of cholesterol and caveolin. Caveolae vesicles traffic virions to the smooth endoplasmic reticulum via a process that is independent of endosomes or lysosomes. SV-40 and echoviruses 1 both use the caveolae pathway to enter cells [17,18]. An alternate pathway for viral entry utilizes clathrin coated pits. In clathrin mediated entry, virions are transported into endosomes and lysosomes. The use of inhibitors that can discriminate between these two pathways has been used to study the mechanisms used by different viral types. These inhibitors include nystatin and methyl-β-cyclodextran which chelate cholesterol resulting in the destabilization of the lipid rafts and prevention of caveolae formation. Similarly, chlorpromazine is an inhibitor of clathrin pathway that prevents endocytosis by preventing the release of clathrin from the endosomes [19,20]. Using these inhibitors together with pseudovirus systems, HPV 16, HPV 33 and BPV 1 have been reported to enter cells by the clathrin mediated pathway [10,11,21]. In contrast, HPV 31 VLPs have been reported to enter cells via the caveolae pathway [21].

In this study we found that the synthesis of wild type HPV 31 L1 and L2 proteins from heterologous expression systems was quite low, however, modification of codons to those more frequently in human genes resulted in high levels of protein synthesis. Our studies further indicate that codon usage itself was probably not the source of the low levels of synthesis but rather was due to the presence of RNA inhibitory elements present in both L1 and L2. These negative regulatory elements are distinct from those previously identified in HPV 16. We then used HPV 31 pseudovirions generated from codon modified capsid proteins to demonstrate that entry occurred through a clathrin-mediated pathway in manner similar to HPV 16.

## Results

### Expression of wild type and codon optimized HPV 31 L1 and L2

**Figure 1:**
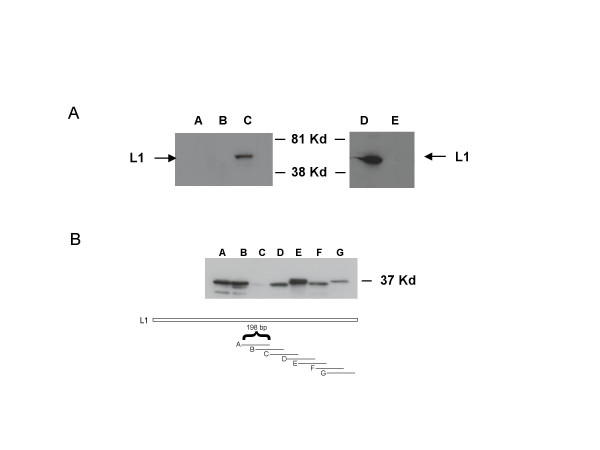
**Western analysis of HPV 31 L1**. (Panel A). Cos-7 cells were transfected with pcDNA 3.1(-) expression vector expressing either wild type HPV 31 L1 or codon optimized HPV 31 L1. After 72 hours, lysates were harvested and analyzed by Western analysis with and antibody to L1. Lane A mock transfected Cos-7 cells. Lane B wild type HPV 31 L1. Lane C Codon optimized HPV 31 L1. Lanes D and E contain L1 peptides tagged with GFP and probed using a GFP antibody. Lane D wild type HPV 31 L1 N-terminus. Lane E wild type HPV 31 L1 C-terminus. (Panel B). Western analysis of in frame fusions to GFP of 198 base pair fragments from the C-terminal domain of wild type L1 following transfection into Cos-7 cells. Each fragment overlapped with the adjacent fragment by 99 base pairs. Following transfection cell extracts were analyzed by Western analysis using a GFP antibody. Lane A plasmid A, GFP fusion to nucleotides 6209–6407 Lane B fragment 2, nucleotides 6308–6506. Lane C fragment 3, nucleotides 6407–6605. Lane D fragment 4, nucleotides 6506–6704. Lane E fragment 5, nucleotides 6605–6803. Lane F fragment 6, nucleotides 6704–6902. Lane G fragment 7, nucleotides 6803–7063. Cartoon shows the fragments of L1 that were fused to GFP and examined in the experiments in panel C. Nucleotide numbers correspond to those in HPV 31 genome.

In order to investigate the mechanisms that regulate HPV 31 capsid assembly and virion entry, we sought to express high levels of capsid proteins using transient transfection assays. Plasmids expressing the HPV 31 wild type L1 gene from SV40 promoters were transfected into Cos-7 cells and after 72 hours, proteins extracts analyzed by Western analysis. In multiple experiments, we failed to observe significant levels of synthesis of L1 (Figure 1). Similar experiments were performed with HPV 31 L2 and yielded comparable low levels of expression (data not shown). Examination of the HPV 31 L1 and L2 coding sequences identified a large number of codons not commonly found in most higher eukaryotic genes and this is similar to that found in other high-risk HPV types [9,22]. Use of these particular codons in HPV 31 results in an unusually low G-C content. It was possible that the low level of expression we observed with HPV 31 capsid proteins was due either to inefficient translation resulting from use of rare codons or to the presence of negative regulatory elements in the RNA [9,22]. We therefore altered the L1 codon usage in a two-step process reasoning that this would increase the levels of protein synthesis by either eliminating any negative regulatory elements or altering translatability. Similar modification of codons in HPV 16 L1 has been reported to result in increased levels of protein expression [9].

First, we altered the coding sequences in HPV 31 L1 to include those codons used frequently in human genes. We then identified codons that had not been changed by this analysis and altered them to the second most frequently used. As a result, by adding this last modification, we introduced at least one base change in almost every codon of L1. The final sequence is referred to as CoOpHPV 31 L1. This optimization of HPV 31 L1 increased the GC content of HPV 31 L1 from 38 to 58 percent. The entire codon optimized sequence for HPV 31 L1 will be deposited in GenBank. Our approach to the codon optimization of HPV 31 L2 was very similar to the method we used for HPV 31 L1. For L2, the GC content was increased from 41 to 59 percent. The entire codon optimized sequence for HPV 31 L2 will also be deposited in GenBank.

### Identification of negative regulatory elements in HPV 31 L1 and L2

**Figure 2:**
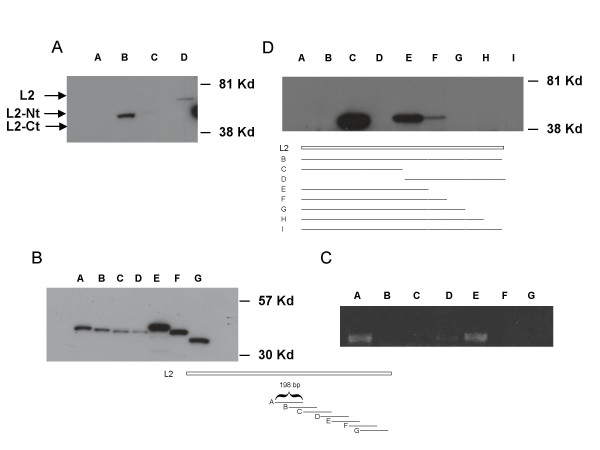
**Western analysis of HPV 31 L2**. Cos-7 cells were transfected with GFP tagged L2 in pcDNA 3.1(-) expression vectors. After 72 hours, lysates were harvested and analyzed by Western blot with an antibody to GFP. (Panel A). Lane A: wild type HPV 31 L2. Lane B: wild type HPV 31 L2 N-terminus. Lane C: wild type HPV 31 L2 C-terminus. Lane D: codon optimized HPV 31 L2. (Panel B). Western analysis of transfections of GFP in frame fusions to 198 base pair fragments of the C-terminal domain of wild type L2. Each fragment overlapped with the adjacent fragment by 99 base pairs. Cell extracts were examined by Western analysis using a GFP antibody. Lane A: fragment A, GFP fusion to nucleotides 4771–4969. Lane B: fragment Bnucleotides 4870–5068. Lane C: fragment C, nucleotides4969–5167. Lane D: fragment D, nucleotides5068–5266. Lane E: fragment E, nucleotides5167–5365. Lane F: fragment F nucleotides 5266–5464. Lane G: fragment G, nucleotides5365–5568. (Panel C). RT-PCR analysis of RNAs isolated from cells transfected with plasmids shown in panel B. Primers to common GFP sequences were used in this analysis. (Panel D) Lane A: mock transfected cells. Lane B: complete wild type HPV 31 L2 fused to GFP nucleotides 4171–5568. Lane C: N-terminal domain wild type L2 (nucleotides 4171–4870) fusion to GFP. Lane D: C-terminal domain wild type L2 (nucleotides 4870–5568) fused to GFP. Lane E: N-terminal domain wild type L2 (nucleotides 4171–4969) fused to GFP. Lane F: N-terminal domain wild type L2 (nucleotides 4171–5068) fused to GFP. Lane G: N-terminal domain wild type L2 (nucleotides 4171–5166) fused to GFP. Lane H: N-terminal domain wild type L2 (nucleotides 4171–5265). Lane I: N-terminal domain wild type L2 (nucleotides 4171–5465). Cartoon identifies fragments of L2 fused to GFP examined in lanesE – I. Nucleotide numbers are those in HPV 31 genome.

We next examined if these changes in codon usage resulted in increased HPV 31 L1 and L2 protein synthesis. Codon optimized HPV 31 L1 and L2 were cloned into SV40 based expression vectors and transfected into Cos-7 cells along with wild type expression vectors. As shown in Figure 1, alteration of the coding sequence resulted in high level of expression of codon optimized L1. Since our L2 antibody had a high background, we tagged wild type and codon optimized L2 with a GFP tag and screened L2-GFP protein levels using an antibody to GFP. Similar to our observations with HPV 31 L1, Cos-7 cells transfected with codon optimized L2 tagged to GFP expressed high levels of protein in comparison to tagged wild type L2 which were not detectable (Figure 2).

Studies were then undertaken to determine if the increase in L1 synthesis was due to more efficient translation due to the use of more commonly used codons or through the elimination of negative regulatory elements present in the L1 RNA. For this analysis we investigated the effect of altering codons on the expression of the N and C-terminal halves of HPV 31 L1. We reasoned that if inefficient synthesis was due to rare codon usage then we should see similarly low levels of expression of both N and C-terminal peptides as seen with the whole L1 protein. Alternatively if RNA negative regulatory elements were present in either half of the protein and these were responsible for low levels of expression then we would see high levels of expression with one truncated protein but not the other. We constructed two plasmids in which the N and C termini of L1 were fused in frame with GFP coding sequences as this allowed for a direct comparison of protein synthesis levels using an antibody to the common tag. The plasmids were transfected into Cos-7 cells and after 72 hours protein extracts were examined by Western analysis. We observed high levels of expression of the fusion of GFP to the wild type HPV 31 L1 N-terminus and barely detectable levels of the C terminal fusion (Figure 1). No such differences in expression levels were seen between codon-modified N and C terminal peptides (data not shown). Since the distribution of codons is similar between the two halves of the L1 protein, our data are consistent with the presence of a negative regulatory element present in the 3' end of the L1 coding sequence. In order to localize this element, we next constructed a series of GFP fusions in which a sequential series of 198 nucleotide DNA fragments from the 3' end of L1 were fused in frame to GFP coding sequences. These plasmids were transfected into cells, extracts isolated after 72 hours and analyzed for GFP protein levels by Western analysis. As shown in Figure 1B, fusion of sequences 6407 to 6605 significantly reduced levels of GFP protein synthesis identifying a major regulatory element in this region. Nucleotide numbers correspond to those in complete HPV 31 genome.

We next investigated if L2 coding sequences exhibited similar properties. Using methods comparable to those for the analysis of L1, N and C terminal fusions of the wild type L2 coding sequences were made to GFP. Expression vectors containing these fusions were then transfected into Cos-7 cells and protein extracts isolated after 72 hours. Western analysis revealed that the N terminal fusion to GFP was synthesized at high levels while the C terminal fusion peptide was not detectable (Figure 2A). We next constructed a series of in frame fusions of 198 base pair fragments isolated from the 3' end of wild type L2 to GFP sequences. Each fragment overlapped with the adjacent fragment by 99 base pairs. As shown in Figure 2B, the three fusions that spanned the region of 699 to 1098 exhibited reduced levels of GFP expression though no single element had as marked an effect as that seen with the fusion of the complete 3' end of the L2 coding sequence. This suggested that there are multiple elements may be present that act cooperatively to reduce levels of L2. We next performed RT-PCR on RNA isolated from the transfections of the various L2 constructs fused to GFP. In the RT-PCR reaction we used primers specific to the GFP portion since GFP was common to all constructs to confirm that the observed reduction was mediated at the RNA level. Consistent with our hypothesis of the presence of an RNA inhibitory element, we noted a corresponding reduction in transcript levels in cells transfected with fusions that spanned 4870 to 5266 (Figure 2C) while transfections with the A and E plasmids exhibited higher message levels. The reduced levels of transcripts observed with the F and G plasmids along with their corresponding high levels of proteins synthesis suggest additional mechanisms may also exist to regulate expression. To provide further support for our hypothesis regarding the presence of multiple elements, we constructed a series of C-terminal deletions of the wild type L2 fusion with GFP and transfected these vectors into Cos-7 cells. As shown in Figure 2D, deletion of sequences 5568 to 5068 restored detectable levels of synthesis, which was further enhanced by deletion to nucleotide 4969. Our results are consistent with the presence of a cooperative series of negative regulatory elements in the 3' end of the L2 coding sequence.

### Assembly of codon optimized HPV 31 pseudovirions

We next sought to use the optimized expression vectors to L1 and L2 to study HPV 31 entry mechanisms. Previous studies have reported the generation of pseudoviruses by the expression of codon optimized HPV 16 L1 and L2 along with reporter plasmids. [9,23]. We utilized a comparable system to study HPV 31 entry by including a plasmid containing a GFP reporter driven by a CMV promoter. The reporter plasmid also contained a region of HPV 31 that encompasses the E1 open reading frame. A similar region in the BPV 1 E1 open reading frame has been reported to enhance the packaging of DNA into viral particles [24]. This reporter plasmid is approximately 8 kb in size and is similar to that of the 7.9 kb HPV 31 genome. Recently smaller vectors of 6.0 kb in size have been suggested to be packaged more efficiently than larger plasmids that approximate the size of the viral genome [23]. However, in our studies, pseudovirions that encapsulate an 8.0 kb plasmid were found to be as infectious as pseudovirions that encapsulate a 6.0 kb vector (data not shown).

In order to produce HPV 31 pseudovirions, we electroporated Cos-7 cells with three separate plasmids consisting of our codon optimized HPV 31 L1 and L2 expressed from the CMV promoter along with the GFP reporter plasmid. We screened for L1 synthesis by Western analysis as well as GFP expression by immunofluoresence and found that expression levels of proteins were highest at 72 h following electroporation (data not shown). Similar effects were seen with human 293TT cells. In our subsequent experiments, we therefore harvested pseudoviruses at 72 hours. Following transfection, cells were harvested, pelleted and resuspended in PBS followed by to 4 to 6 rounds of freeze-thaw. The lysed cells were then treated with DNase 1 and incubated for 1 h at 37°C. The treated lysate solution was brought to a final concentration of 0.8 M NaCl and incubated for 1–3 h at 37°C. The lysate was then centrifuged at low speed, which removes the cellular debris but allows the pseudovirions to remain in the supernatant. Previous studies using similar protocols have shown HPV 16 pseudovirions are retained in the supernatant [23].

### Analysis of entry pathway used by HPV 16 and 31 pseudovirions

**Figure 3:**
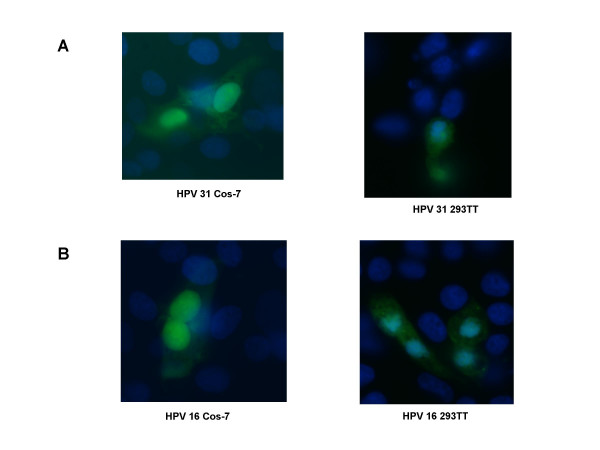
**Infection of Cos-7 and 293 TT cells by HPV 31 and HPV 16 pseudovirions**. (A). HPV 31 pseudovirions were used to infect Cos-7 cells (left panel) or 293 TT cells (right panel). (B). HPV 16 pseudovirions were used to infect Cos-7 cells or 293 TT cells (right panel). After 24 hours, the media was changed. The infection was allowed to proceed for a total of 72 hours where maximal GFP expression occurred. The cells were fixed and stained with DAPI. Shown is a representative field of infected cells detected by GFP fluorescence.

We next used our HPV 31 pseudovirions to examine the parameters that influence entry. HPV 31 pseudovirion preparations were added to sub-confluent, monolayer cultures of Cos-7 or 293TT cells and incubated for various periods of time. The cells were then fixed and analyzed by fluorescence microscopy. We observed initial fluorescence at approximately 36 hours post infection that peaked at 72 hours. Figure 3 shows representative GFP expression in Cos-7 and 293 TT cells that are positive for HPV 31 pseudovirion infection at 72 hours post-infection. GFP expression occurs only if the genome has been transported to the nucleus and the reporter plasmid has been transcribed [25]. Production of HPV 31 pseudovirions and infectivity was found to be significantly higher in 293 TT cells than Cos-7 cells.

In order to compare the different entry pathways used by various HPV types, we included control infections using similarly isolated preparations of HPV 16 pseudoviruses. To generate HPV 16 pseudoviruses, we transfected expression vectors for codon optimized HPV 16 L1 and L2 [9] from Martin Mueller's lab along with the same GFP reporter plasmid into Cos-7 or 293 TT cells. HPV 16 pseudovirions were isolated by the same procedure used for HPV 31 pseudoviruses and added to sub-confluent Cos-7 or 293 TT cells. After 72 hours, the cells were fixed and analyzed by fluorescence microscopy. We observed the first GFP signal at 36 hours after infection with a time course that was similar to our HPV 31 infections (data not shown). Again as we found with HPV 31, production of HPV 16 pseudovirions and infectivity was significantly higher in 293 TT cells than Cos-7 cells.

HPV 31 has been previously reported to enter cells through the caveolae pathway while other high-risk HPV types such as HPV 16 and 33 utilize clathrin coated pits to transit into the cell [11,21]. We sought to investigate the process by which HPV 31 pseudoviruses entered cells through the use of inhibitors of caveolae/lipid rafts such as nystatin and methyl-β-cyclodextrin as well as chlorpromazine which impedes clathrin entry [26-28]. In our studies we used the same concentrations used in the previous experiments with pseudoviruses.

**Figure 4:**
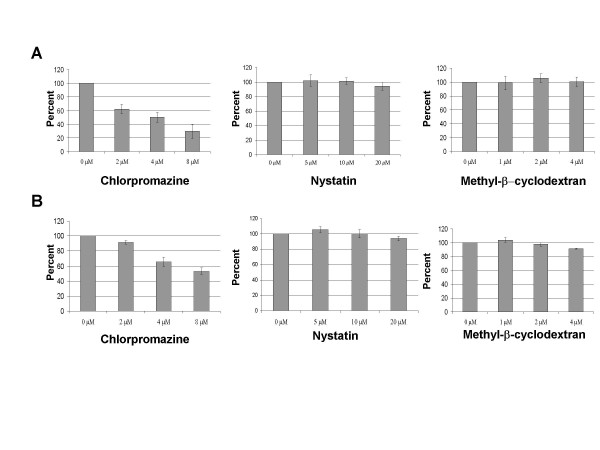
**Inhibition of HPV 16 pseudovirion infection**. (A) Cos-7 cells or (B) 293 TT cells were treated with inhibitors chlorpromazine, nystatin or methyl-β-cyclodextran at the concentrations shown for 30 min prior to infection. The cells were infected over night with HPV 16 pseudovirions. Media was changed at 24 hours, fresh media and drug was added. The infection was allowed to continue for an additional 48 hours for a total of 72 hours. Percentage GFP positive cells relative to control is shown. Each panel is an average of 3 experiments.

**Figure 5:**
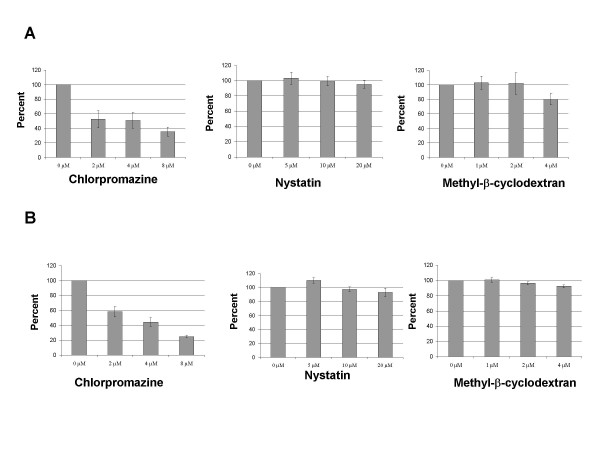
**Inhibition of HPV 31 pseudovirion infection**. (A) Cos-7 cells or (B) 293 TT cells were treated with inhibitors chlorpromazine, nystatin or methyl-β-cyclodextran at the concentrations shown for 30 min prior to infection. The cells were infected over night with HPV 31 pseudovirions. Media was changed at 24 hours, fresh media and drug was added. The infection was allowed to continue for an additional 48 hours for a total of 72 hours. Percentage GFP positive cells relative to control is shown. Each panel is an average of 3 experiments.

We next wanted to confirm, using our experimental protocols, that HPV 16 pseudoviruses enter cells by the clathrin mediated pathway as has been reported [10-12]. In these experiments we added the inhibitors (either nystatin, methyl-β-cyclodextrin or chlorpromazine) 30 minutes prior to infection and maintained for 72 h. In Figure 4, averaged results from three experiments are shown in which Cos-7 and 293 TT cells were infected with HPV 16 pseudovirions in the presence or absence of inhibitors. Similar results were seen in multiple experiments. As the concentration of chlorpromazine was increased, we observed a significant decrease in HPV 16 pseudovirion entry (Figure 4). In contrast, the presence of nystatin and methyl-β-cyclodextrin exhibited no effect on HPV 16 pseudovirion infectivity. These concentrations have been reported to inhibit the infection by BPV-1, HPV 33 and EBV [10,11,21,26]. We next performed identical experiments with HPV 31 pseudoviruses and the results are shown in Figure 5. As we observed with HPV 16 pseudoviruses, increasing concentrations of chlorpromazine decreased HPV 31 pseudovirion entry in a dose-dependent manner into either Cos-7 or 293 TT cells. The concentrations of inhibitors used in Figure 5 had no effect on cell viability but higher concentrations of chlorpromazine were observed to be toxic to the Cos-7 and 293 TT cells (data not shown). As shown in Figure 5, no decrease on HPV 31 pseudovirion infectivity was observed as nystatin or methyl-β-cyclodextrin levels were increased. Interestingly, at intermediate concentrations of nystatin or methyl-β-cyclodextrin, we observed a reproducible increase in infectivity over untreated cells. In addition we observed that treatment with the drugs was reversible, as infections in the presence of chlorpromazine for 2–6 hours followed by a wash with fresh media and incubation for a total of 72 hours had negligible effect on infectivity (data not shown). Our results suggest a role for clathrin-mediated entry of HPV 16 and 31 pseudovirions in the entry of human and monkey cells.

## Discussion and conclusion

In this study we have examined the regulation of HPV 31 capsid protein synthesis and the mechanism of viral entry. The expression of capsid proteins of papillomaviruses occurs late in the viral life cycle in terminally differentiated epithelial cells. This restriction has limited the full understanding of the papillomavirus life cycle. In transient overexpression assays using heterologous promoter constructs, we observed that the levels of synthesis of wild type HPV 31 L1 and L2 protein were so low that they were not detectable by standard Western analysis. We reasoned that this low level synthesis was the result of the presence in L1 and L2 of rare codons that are infrequently found in mammalian genes. When these codons were modified to ones more commonly found in human coding sequences, the levels of synthesis of the capsid proteins increased significantly. The presence of infrequently used codons does not appear alone to be sufficient to limit expression of the late proteins as a similar frequency of rare codon usage is found in other HPV 31 ORF's including those for E1, E2, E5, E6 and E7. Utilization of infrequently used codons could contribute to low levels of viral protein translation and so aid in escaping immune detection. However, since the capsid proteins are only synthesized in highly differentiated cells, which are located at a distance from most antigen presenting cells this does not seem to be a likely explanation. An alternate hypothesis is that the tRNAs encoding these infrequently used codons are more abundant in differentiating keratinocytes, but there is no convincing evidence for such a cell type specific distribution of tRNAs in human keratinocytes.

Our studies provide evidence that utilization of rare codons in HPV 31 results from the presence of negative regulatory elements in the mRNAs themselves that likely regulate stability of late messages. In HPV 16, a negative regulatory element was identified at the 5'end of L1 and regulates usage of the upstream splice site [29-31]. The negative element we identified in HPV 31 is located at the 3' end of the L1 open reading frame and did not function by altering splicing. An additional series of negative elements have been identified in HPV 16 L2 but in contrast to our studies with HPV 31, these elements are located at the 5' end as well as in the middle of L2 [36]. In HPV 31, we found a series of cooperative elements localized to the 3' end of the open reading frame. It is unclear whether the HPV 31 elements may act in a similar manner to those of HPV 16. It is also possible that an unequal distribution of a small set of rare codons that are localized to these elements contribute to reduced levels of synthesis. Further analysis will require alteration of these elements in the context of the complete HPV genome and screening for effects on the viral life cycle following differentiation.

The RNA inhibitory elements we identified in the L1 and L2 genes of HPV 31 may act to regulate the stability or translatability of late mRNAs. These elements may be recognized by trans acting factors that are modulated upon differentiation to allow for capsid proteins synthesis in suprabasal cells. Several cellular factors have been reported to negatively regulate the stability of capsid transcripts of various papillomaviruses including heterogeneous ribonucleoprotein K (hnRNP K), hnRNP C, poly (rC) binding-protein (PCBP), U1 snRNP and HuR [29-35]. Similar to our studies on HPV 31, the optimization of capsid sequences for HPV 11, 16 and BPV 1 have been shown to increase levels of protein synthesis and may contain similar elements [9,36,37]. Our studies are consistent with important roles for these negative regulatory elements in the pathogenesis of high risk HPVs.

The HPV 31 pseudovirions synthesized using the codon optimized capsid proteins were then used together with a GFP reporter plasmid to investigate the mechanisms by which these viral types enter cells. Previous studies used HPV 31 VLPs to which DNA reporter plasmids had been adhered on the external surface to examine pathways by which HPVs enter cells [21]. Using inhibitors of the clathrin-mediated and caveolae pathways, it was reported that HPV 16 entered cells by the clathrin-mediated mechanism while HPV 31 entered by caveolae. The ability of HPV 16 pseudovirions to enter cells by a clathrin-mediated pathway had been also reported by John Schiller's group [10]. In our study, we examined entry using our HPV 31 pseudovirions in the presence of the caveolae inhibitors nystatin and methyl-β-cyclodextrin along with chlorpromazine, which inhibits clathrin mediated uptake. We observed that like HPV 16, HPV 31 entry was inhibited only by chlorpromazine indicating entry occurs via the clathrin-mediated pathway. We suspect that the previous use of VLP's to which reporter plasmids were coupled on the external surface may have altered the pathway by which HPV 31 normally enters cells. Our data are consistent with the idea that the oncogenic HPV types enter cells by a common mechanism. We cannot, however, exclude the possibility that there are cell type specific differences in the mechanisms by which HPV 's enter cells and that there might be cells where HPV 31 uses caveolae to enter cells. The identification of a common pathway used by high risk HPV types suggests that antivirals could be developed that target clathrin-mediated entry to block infections by these highly pathogenic viruses.

## Methods

### Codon optimization of HPV 31 L1 and L2

To codon optimize L1 and L2, the sequence was recoded to reflect the most common codons used by human cells. This sequence was then aligned with the wild type sequence using Lasergene MegAlign (Madison Wi, 53715). Any identical codons identified in the alignment were changed to a different codon, usually the most common codon for that given amino acid. The sequences were synthesized by DNA 2.0 (Menlo Park, CA 94025) and cloned into a pDrive cloning vector (Qiagen, Hilden Germany).

### Plasmids constructs

The reporter plasmid pHcDNA8k GFP was created by cloning the green fluorescent protein (GFP) gene (Clontech Mountain View, CA 94043) into Bam H1 and Hind III sites of pcDNA 3.1 (-) (Invitrogen Carlsbad, California 92008). In addition a fragment of HPV 31 E1 (base pairs 1048–2880) were cloned into the unique Bgl II site of pcDNA 3.1 (-). Codon optimized HPV 31 L1 and L2 (pHcDCoOp31L1 and pHcDCoOp31L2) and wild type HPV 31 L1 and L2 (pHcDWT31L1 and pHcDWT31L2) were cloned into pcDNA 3.1 (-) at Xho1 and Eco R1 sites. These vectors contain an SV40 origin which allows for replication in cells that express T-antigen. All primers were purchased from IDT (Coralville, IA 52241). HPV 16 L1 and L2 constructs were generously provided by Martin Muller Heidelberg, Germany.

### Cell lines and transfections

Cos-7 cells were maintained in Dulbecco's modified Eagle medium (DMEM) (Invitrogen) and supplemented with 10 percent fetal bovine serum (FBS) (Hyclone Logan Utah, 84321). 293TT cells (generously provided by John Schiller, NIH) were maintained in DMEM and 10 percent FBS supplemented with 1 percent non essential amino acids (Invitrogen), 1 percent Glutamax 1 (Invitrogen) and cultured with hygromycin B (Roche). Cells were transfected using a BTX ECM 630 Electro Cell Manipulator (BTX San Diego Ca 92121) 170 V and 950 μF capacitance. Typically 1–9 μg of DNA was electroporated along with 1–2 million cells in 300 μl DMEM and 10 percent FBS using a 0.4 cm BioRad cuvette (BioRad Hercules Ca 94547).

### Western blot analysis and RT-PCR

Cos-7 cells were harvested 72 hours post transfection and whole cell extracts were prepared using NP-40 lysis buffer (150 mM NaCl, 50 mM Tris-HCl [pH 8], 5 mM EDTA [pH 8], 0.5 mM dithiothreitol, 100 mM sodium fluoride, 200 μM sodium-orthovanadate, 0.5% NP-40, 1 mM phenylmethylsulfonyl fluoride) containing a cocktail of protease inhibitors (Complete, Mini; Roche Diagnostic). Cell lysates were cleared of insoluble material by centrifugation at 12,000 RPM for 10 minutes. The lysates were quantitated with the Bradford assay (Bio-Rad). Proteins were separated using a 10 percent SDS-polyacrylamide gel and transferred to a polyvinylidene difluoride membrane (Immobilon-P; Millipore, Bedford, Mass.). The membrane was blocked in wash solution containing 5% nonfat dry milk, 0.1% Tween 20 in PBS. HPV L1 (HPV16.D9) monoclonal antibody was a generous gift form Neil Christensen, Pennsylvania State University College of Medicine, Hershey, PA 17033. Living Colors GFP monoclonal antibody (JL-8) (Clontech). Sheep anti-mouse HRP (NA931V) and sheep anti-mouse FITC (N1031V) monoclonal antibodies were from Amersham Biosciences UK. Specific proteins were visualized via enhanced chemiluminescence (Amersham-Pharmacia).

Cos-7 cells were electroporated with GFP tagged constructs used in Figures 1B and 2B. Total cellular RNA was isolated using SV Total RNA Isolation System (Promega) as per manufacturer instructions. 1 ug of total RNA was used in the RT-PCR reactions (Reverse Transcriptase System, Promega). To amplify the RT product we used primers to GFP since GFP was common to all constructs used in these reactions. As a control, reactions were performed in the absence RT to control for plasmid contamination.

### Fluorescent microscopy

Cos-7 cells were electroporated with plasmids or infected with pseudovirions and grown on glass cover slips (Corning). After 72 hours the cells were washed in PBS, fixed in 100 percent Methanol, stained with 10 μg/ml DAPI (Sigma) and allowed to air dry. The cover slips were then coated with Vectashield, Vector Laboratories, Burlingame Ca. 94010.

### Preparation of HPV 31 and 16 pseudovirions and infection of target cells

Cos-7 cells or 293TT cells [23] were electroporated with L1, L2 and the reporter plasmid as described above. The cells were allowed to grow for 72 hours and harvested. Our harvesting method is a modification described by Buck et al., [23]. Transfected Cos-7 or 293TT cells were washed with PBS and trypsinsized (Invitrogen). The trypsin was neutralized with DMEM plus FBS and centrifuged at 190 xg for 4 min at room temperature. The pellet was then washed with PBS and centrifuged as above. The pellet was resuspended in PBS supplemented with Complete Mini protease inhibitor (Roche, Mannheim, Germany) and subjected to 3–6 rounds of freeze-thawing (ethanol-dry ice transferred to 37°C water bath). MgCl_2 _was added to the suspension to a final concentration of 2.5 mM. To remove any free reporter plasmid the pseudovirions were treated with 5 units of DNAse 1 (New England Biolabs, Beverly, Ma) for 1 hour at 37°C. NaCl was added to the digested lysate at a final concentration of 0.8 M. The lysate was then incubated at room temperature for 1–3 hours followed by a low speed centrifugation (2000 × g for 10 minutes). The isolated pseudovirions were maintained on ice prior to infection assays. Cos-7 or 293TT cells were infected with 1/10 volume of the pseudovirion supernatant. At 24 hours post infection, the media was replaced and the infection continued for an additional 48 hours. GFP expression was observed at 36 hours with a maximal expression at 72 hours. Nystatin (Sigma), methyl-β-cyclodextrin (Sigma) and chlorpromazine (Alexis, San Diego Ca. 92121) were used at the concentrations indicated. Nystatin, methyl-β-cyclodextrin and chlorpromazine were added to Cos-7 or 293TT cells 30 minutes prior to infection. At 24 hours the media was changed and fresh drug was added and maintained for an additional 48 hours.

## Abbreviations

none

## Competing interests

The author(s) declare that they have no competing interests.

## Authors' contributions

PLH performed all the experiments, worked on the design of the project as well as on the drafting of the manuscript. LAL participated in the design of the project and coordination as well as helped to draft the manuscript.
